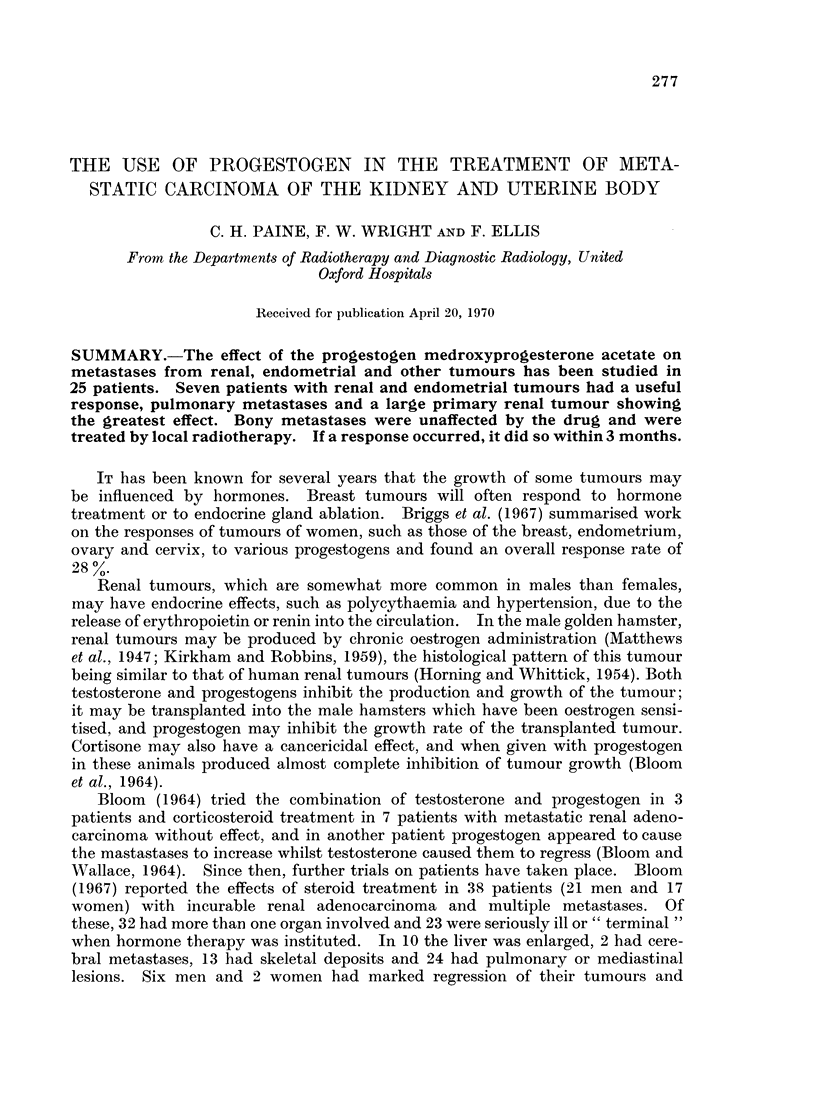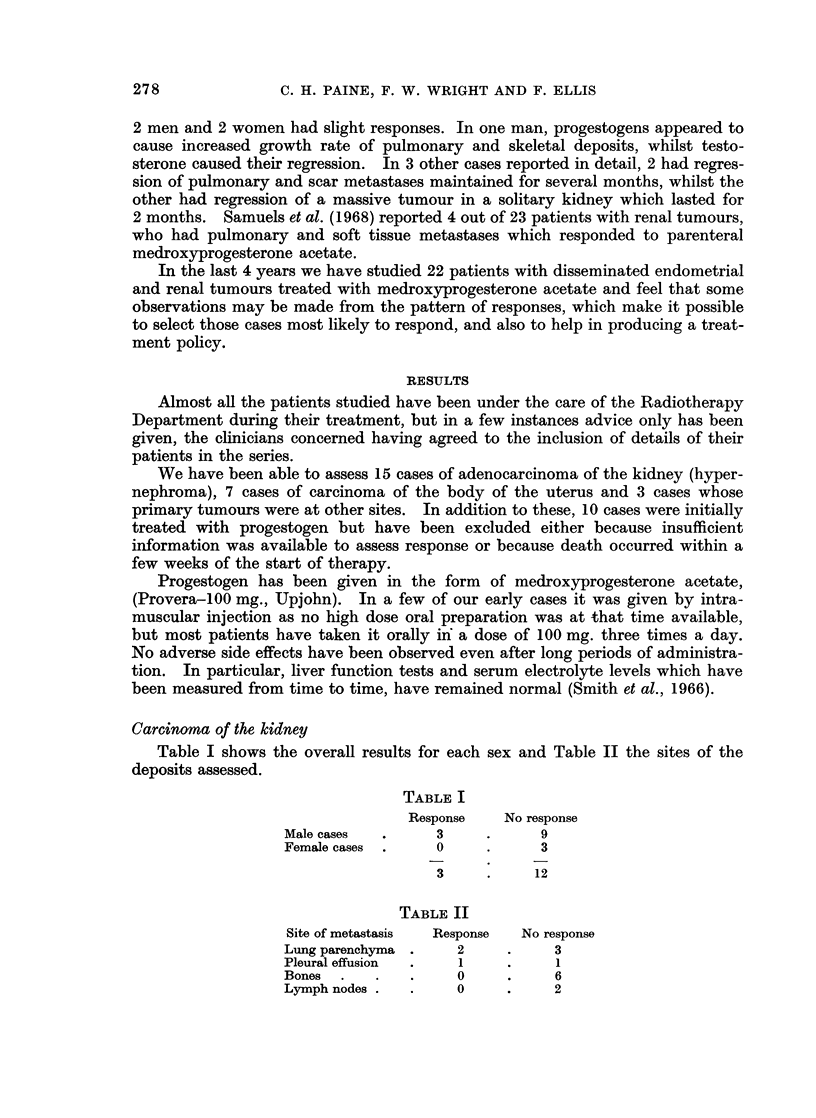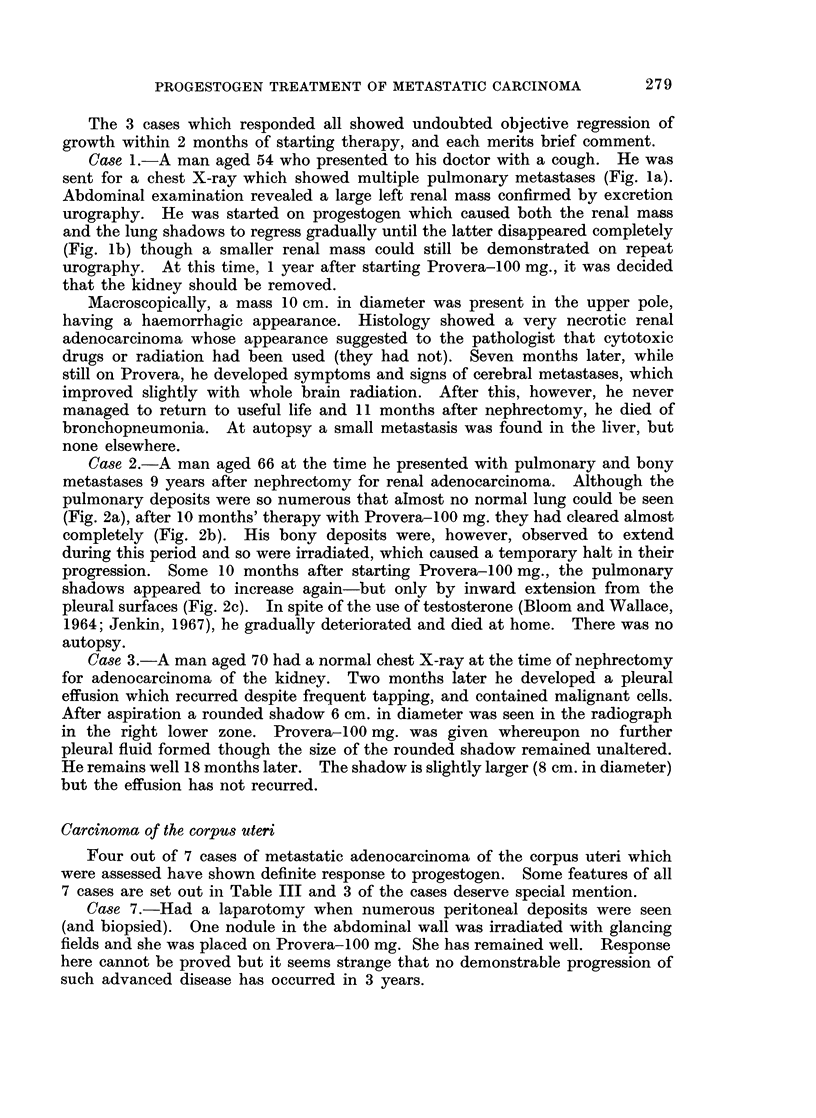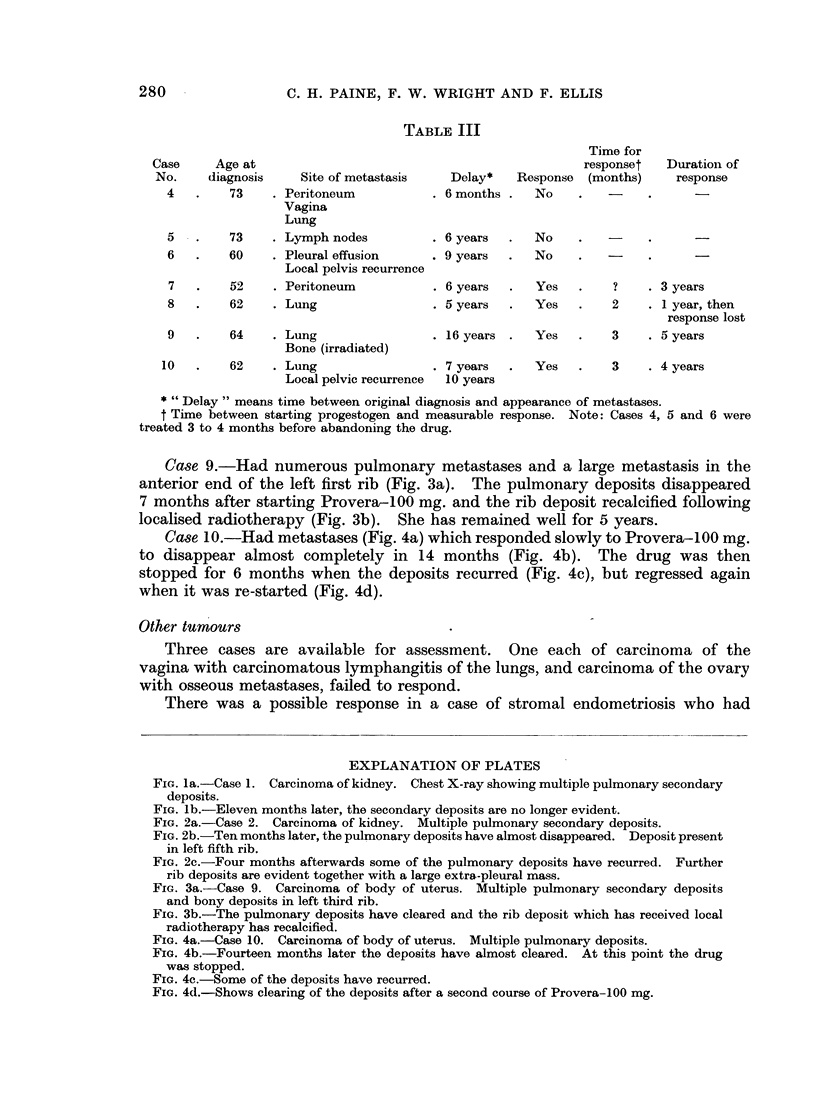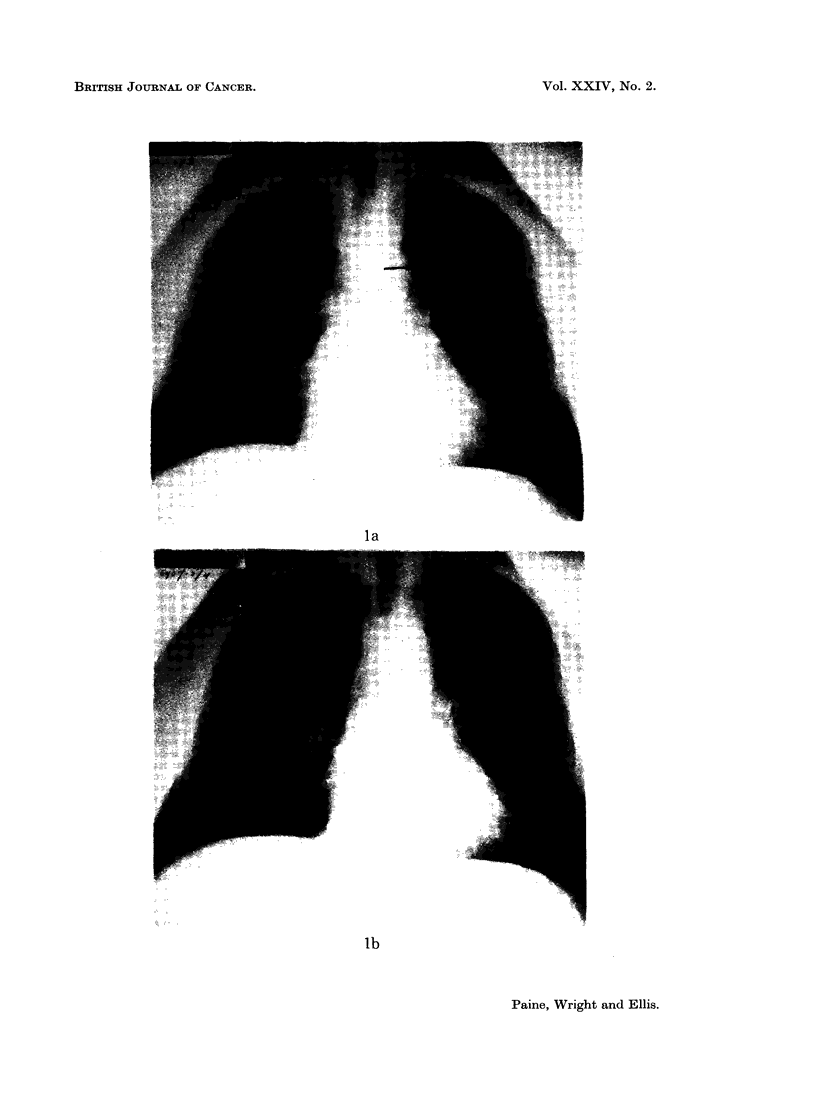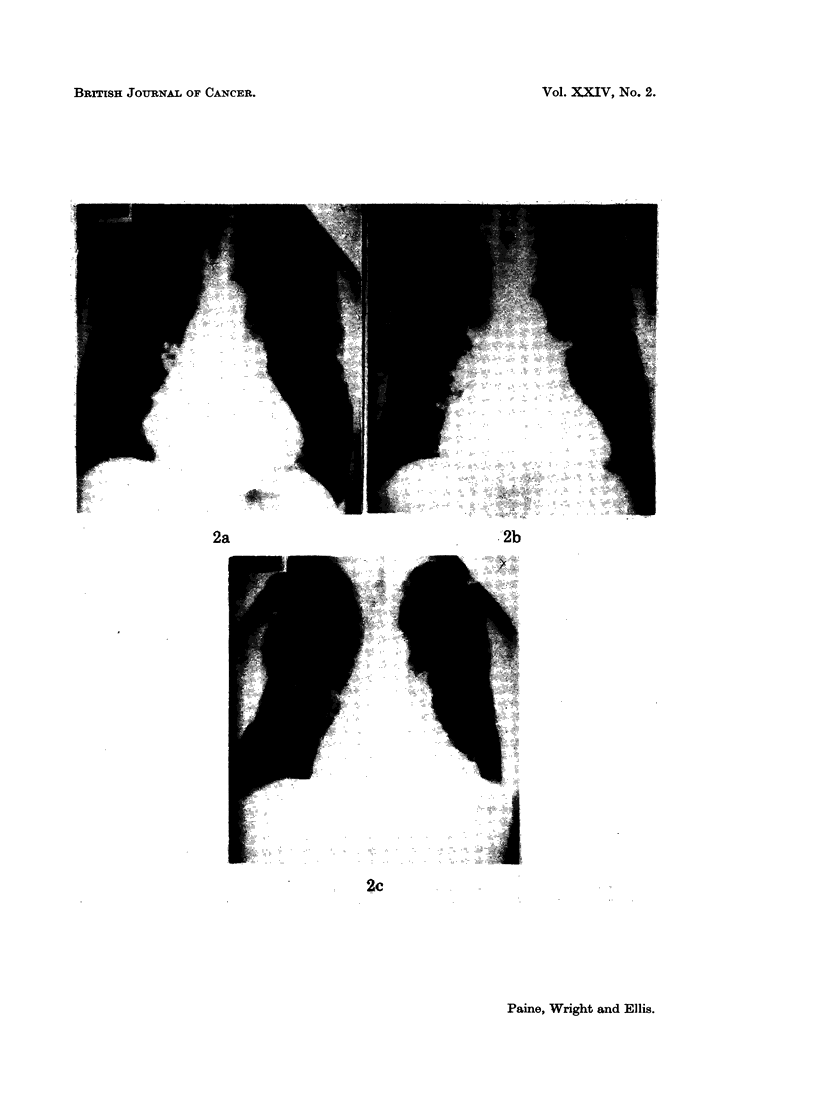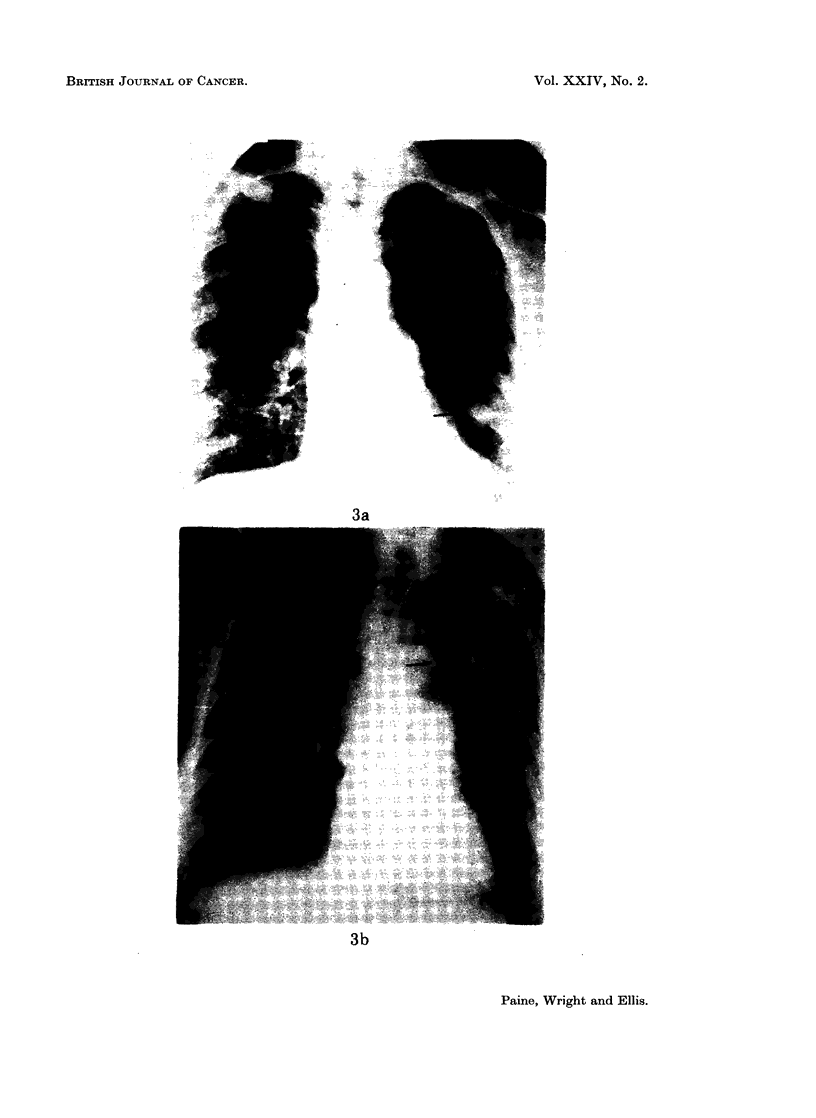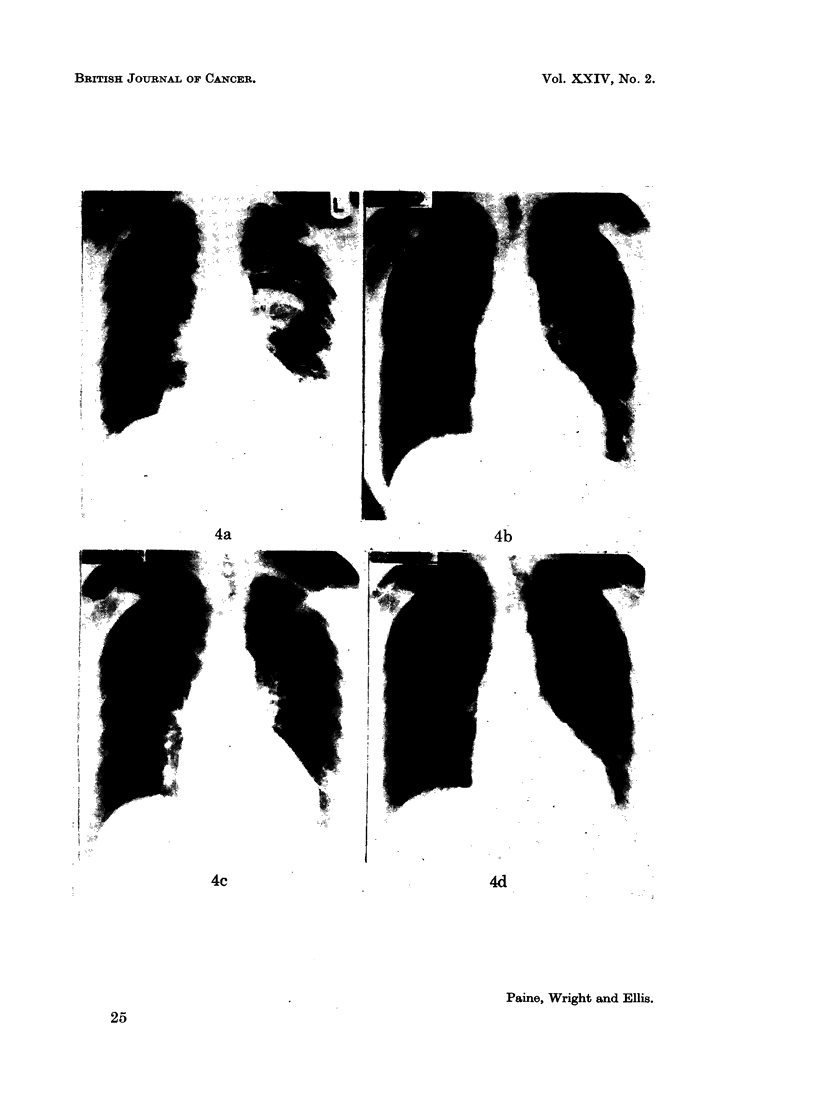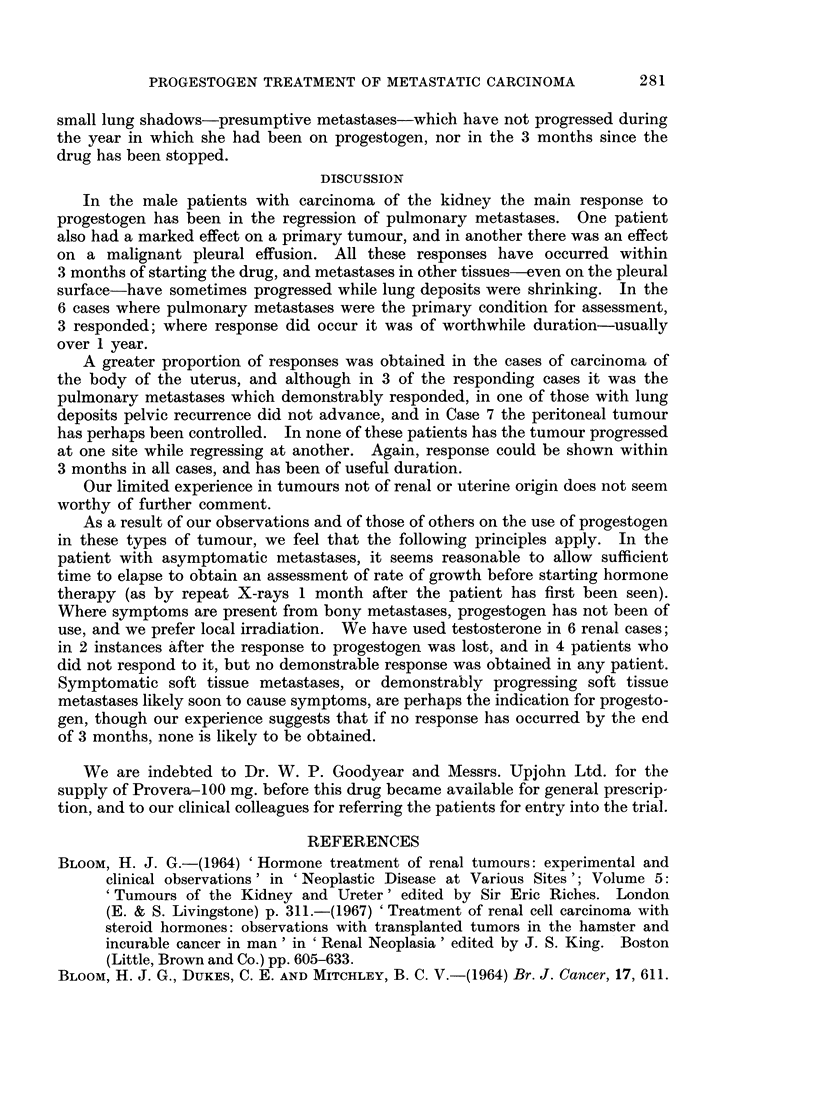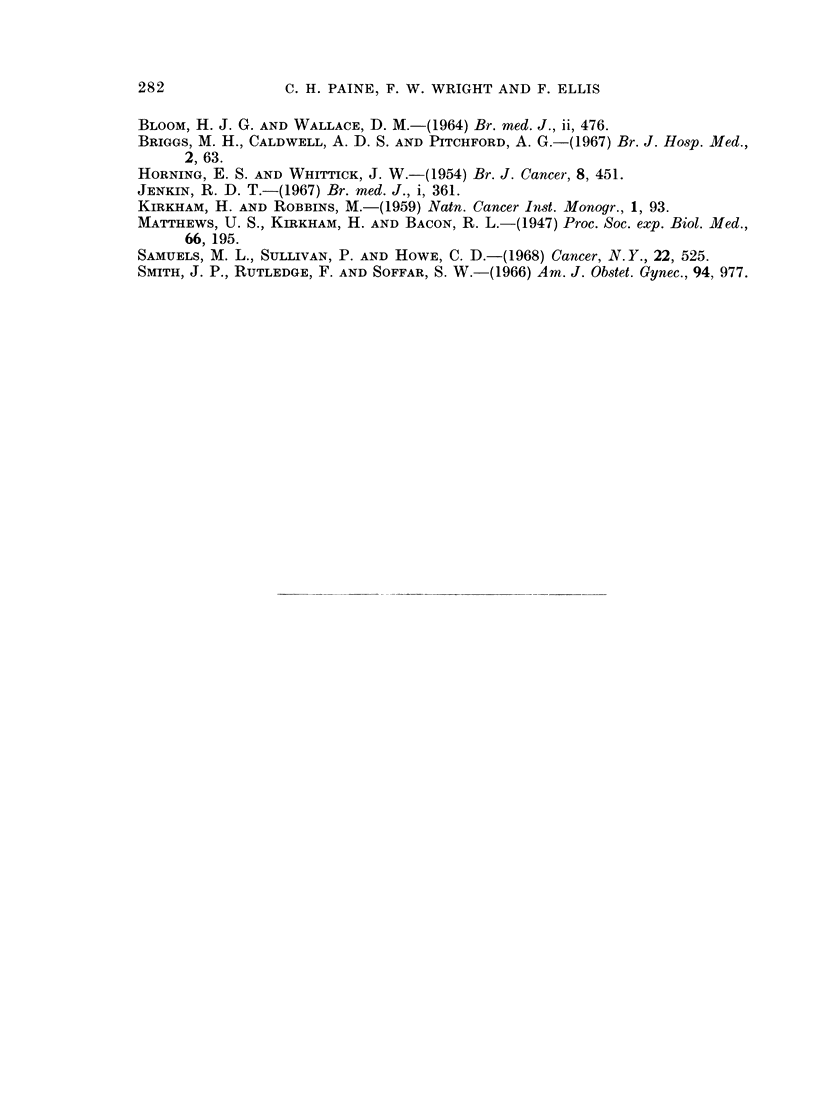# The Use of Progestogen in the Treatment of Metastatic Carcinoma of the Kidney and Uterine Body

**DOI:** 10.1038/bjc.1970.31

**Published:** 1970-06

**Authors:** C. H. Paine, F. W. Wright, F. Ellis

## Abstract

**Images:**


### THE USE OF PROGESTOGEN IN THE TREATMENT OF METASTATIC CARCINOMA OF THE KIDNEY AND UTERINE BODY

C. H. PAINE, F. W. WRIGHT AND F. ELLIS

*From the Departments of Radiotherapy and Diagnostic Radiology, United Oxford Hospitals*

Received for publication April 20, 1970

**SUMMARY.—The effect of the progestogen medroxyprogesterone acetate on metastases from renal, endometrial and other tumours has been studied in 25 patients. Seven patients with renal and endometrial tumours had a useful response, pulmonary metastases and a large primary renal tumour showing the greatest effect. Bony metastases were unaffected by the drug and were treated by local radiotherapy. If a response occurred, it did so within 3 months.**

It has been known for several years that the growth of some tumours may be influenced by hormones. Breast tumours will often respond to hormone treatment or to endocrine gland ablation. Briggs *et al.* (1967) summarised work on the responses of tumours of women, such as those of the breast, endometrium, ovary and cervix, to various progestogens and found an overall response rate of 28%.

Renal tumours, which are somewhat more common in males than females, may have endocrine effects, such as polycythaemia and hypertension, due to the release of erythropoietin or renin into the circulation. In the male golden hamster, renal tumours may be produced by chronic oestrogen administration (Matthews *et al.*, 1947; Kirkham and Robbins, 1959), the histological pattern of this tumour being similar to that of human renal tumours (Horning and Whittick, 1954). Both testosterone and progestogens inhibit the production and growth of the tumour; it may be transplanted into the male hamsters which have been oestrogen sensitised, and progestogen may inhibit the growth rate of the transplanted tumour. Cortisone may also have a cancericidal effect, and when given with progestogen in these animals produced almost complete inhibition of tumour growth (Bloom *et al.*, 1964).

Bloom (1964) tried the combination of testosterone and progestogen in 3 patients and corticosteroid treatment in 7 patients with metastatic renal adenocarcinoma without effect, and in another patient progestogen appeared to cause the mastastases to increase whilst testosterone caused them to regress (Bloom and Wallace, 1964). Since then, further trials on patients have taken place. Bloom (1967) reported the effects of steroid treatment in 38 patients (21 men and 17 women) with incurable renal adenocarcinoma and multiple metastases. Of these, 32 had more than one organ involved and 23 were seriously ill or " terminal " when hormone therapy was instituted. In 10 the liver was enlarged, 2 had cerebral metastases, 13 had skeletal deposits and 24 had pulmonary or mediastinal lesions. Six men and 2 women had marked regression of their tumours and

2 men and 2 women had slight responses. In one man, progestogens appeared to cause increased growth rate of pulmonary and skeletal deposits, whilst testosterone caused their regression. In 3 other cases reported in detail, 2 had regression of pulmonary and scar metastases maintained for several months, whilst the other had regression of a massive tumour in a solitary kidney which lasted for 2 months. Samuels *et al.* (1968) reported 4 out of 23 patients with renal tumours, who had pulmonary and soft tissue metastases which responded to parenteral medroxyprogesterone acetate.

In the last 4 years we have studied 22 patients with disseminated endometrial and renal tumours treated with medroxyprogesterone acetate and feel that some observations may be made from the pattern of responses, which make it possible to select those cases most likely to respond, and also to help in producing a treatment policy.

### RESULTS

Almost all the patients studied have been under the care of the Radiotherapy Department during their treatment, but in a few instances advice only has been given, the clinicians concerned having agreed to the inclusion of details of their patients in the series.

We have been able to assess 15 cases of adenocarcinoma of the kidney (hypernephroma), 7 cases of carcinoma of the body of the uterus and 3 cases whose primary tumours were at other sites. In addition to these, 10 cases were initially treated with progestogen but have been excluded either because insufficient information was available to assess response or because death occurred within a few weeks of the start of therapy.

Progestogen has been given in the form of medroxyprogesterone acetate, (Provera–100 mg., Upjohn). In a few of our early cases it was given by intramuscular injection as no high dose oral preparation was at that time available, but most patients have taken it orally in a dose of 100 mg. three times a day. No adverse side effects have been observed even after long periods of administration. In particular, liver function tests and serum electrolyte levels which have been measured from time to time, have remained normal (Smith *et al.*, 1966).

### *Carcinoma of the kidney*

Table I shows the overall results for each sex and Table II the sites of the deposits assessed.

TABLE I

| | Response | No response |
|---|---|---|
| Male cases | 3 | 9 |
| Female cases | 0 | 3 |
| | 3 | 12 |

TABLE II

| Site of metastasis | Response | No response |
|---|---|---|
| Lung parenchyma | 2 | 3 |
| Pleural effusion | 1 | 1 |
| Bones | 0 | 6 |
| Lymph nodes | 0 | 2 |

The 3 cases which responded all showed undoubted objective regression of growth within 2 months of starting therapy, and each merits brief comment.

*Case* 1.—A man aged 54 who presented to his doctor with a cough. He was sent for a chest X-ray which showed multiple pulmonary metastases (Fig. 1a). Abdominal examination revealed a large left renal mass confirmed by excretion urography. He was started on progestogen which caused both the renal mass and the lung shadows to regress gradually until the latter disappeared completely (Fig. 1b) though a smaller renal mass could still be demonstrated on repeat urography. At this time, 1 year after starting Provera–100 mg., it was decided that the kidney should be removed.

Macroscopically, a mass 10 cm. in diameter was present in the upper pole, having a haemorrhagic appearance. Histology showed a very necrotic renal adenocarcinoma whose appearance suggested to the pathologist that cytotoxic drugs or radiation had been used (they had not). Seven months later, while still on Provera, he developed symptoms and signs of cerebral metastases, which improved slightly with whole brain radiation. After this, however, he never managed to return to useful life and 11 months after nephrectomy, he died of bronchopneumonia. At autopsy a small metastasis was found in the liver, but none elsewhere.

*Case* 2.—A man aged 66 at the time he presented with pulmonary and bony metastases 9 years after nephrectomy for renal adenocarcinoma. Although the pulmonary deposits were so numerous that almost no normal lung could be seen (Fig. 2a), after 10 months' therapy with Provera–100 mg. they had cleared almost completely (Fig. 2b). His bony deposits were, however, observed to extend during this period and so were irradiated, which caused a temporary halt in their progression. Some 10 months after starting Provera–100 mg., the pulmonary shadows appeared to increase again—but only by inward extension from the pleural surfaces (Fig. 2c). In spite of the use of testosterone (Bloom and Wallace, 1964; Jenkin, 1967), he gradually deteriorated and died at home. There was no autopsy.

*Case* 3.—A man aged 70 had a normal chest X-ray at the time of nephrectomy for adenocarcinoma of the kidney. Two months later he developed a pleural effusion which recurred despite frequent tapping, and contained malignant cells. After aspiration a rounded shadow 6 cm. in diameter was seen in the radiograph in the right lower zone. Provera–100 mg. was given whereupon no further pleural fluid formed though the size of the rounded shadow remained unaltered. He remains well 18 months later. The shadow is slightly larger (8 cm. in diameter) but the effusion has not recurred.

### *Carcinoma of the corpus uteri*

Four out of 7 cases of metastatic adenocarcinoma of the corpus uteri which were assessed have shown definite response to progestogen. Some features of all 7 cases are set out in Table III and 3 of the cases deserve special mention.

*Case* 7.—Had a laparotomy when numerous peritoneal deposits were seen (and biopsied). One nodule in the abdominal wall was irradiated with glancing fields and she was placed on Provera–100 mg. She has remained well. Response here cannot be proved but it seems strange that no demonstrable progression of such advanced disease has occurred in 3 years.

TABLE III

| Case No. | Age at diagnosis | Site of metastasis | Delay* | Response | Time for response† (months) | Duration of response |
|---|---|---|---|---|---|---|
| 4 | 73 | Peritoneum<br>Vagina<br>Lung | 6 months | No | — | — |
| 5 | 73 | Lymph nodes | 6 years | No | — | — |
| 6 | 60 | Pleural effusion<br>Local pelvis recurrence | 9 years | No | — | — |
| 7 | 52 | Peritoneum | 6 years | Yes | ? | 3 years |
| 8 | 62 | Lung | 5 years | Yes | 2 | 1 year, then response lost |
| 9 | 64 | Lung<br>Bone (irradiated) | 16 years | Yes | 3 | 5 years |
| 10 | 62 | Lung<br>Local pelvic recurrence | 7 years<br>10 years | Yes | 3 | 4 years |

* " Delay " means time between original diagnosis and appearance of metastases.
† Time between starting progestogen and measurable response. Note: Cases 4, 5 and 6 were treated 3 to 4 months before abandoning the drug.

*Case* 9.—Had numerous pulmonary metastases and a large metastasis in the anterior end of the left first rib (Fig. 3a). The pulmonary deposits disappeared 7 months after starting Provera–100 mg. and the rib deposit recalcified following localised radiotherapy (Fig. 3b). She has remained well for 5 years.

*Case* 10.—Had metastases (Fig. 4a) which responded slowly to Provera–100 mg. to disappear almost completely in 14 months (Fig. 4b). The drug was then stopped for 6 months when the deposits recurred (Fig. 4c), but regressed again when it was re-started (Fig. 4d).

*Other tumours*

Three cases are available for assessment. One each of carcinoma of the vagina with carcinomatous lymphangitis of the lungs, and carcinoma of the ovary with osseous metastases, failed to respond.

There was a possible response in a case of stromal endometriosis who had

EXPLANATION OF PLATES

FIG. 1a.—Case 1. Carcinoma of kidney. Chest X-ray showing multiple pulmonary secondary deposits.
FIG. 1b.—Eleven months later, the secondary deposits are no longer evident.
FIG. 2a.—Case 2. Carcinoma of kidney. Multiple pulmonary secondary deposits.
FIG. 2b.—Ten months later, the pulmonary deposits have almost disappeared. Deposit present in left fifth rib.
FIG. 2c.—Four months afterwards some of the pulmonary deposits have recurred. Further rib deposits are evident together with a large extra-pleural mass.
FIG. 3a.—Case 9. Carcinoma of body of uterus. Multiple pulmonary secondary deposits and bony deposits in left third rib.
FIG. 3b.—The pulmonary deposits have cleared and the rib deposit which has received local radiotherapy has recalcified.
FIG. 4a.—Case 10. Carcinoma of body of uterus. Multiple pulmonary deposits.
FIG. 4b.—Fourteen months later the deposits have almost cleared. At this point the drug was stopped.
FIG. 4c.—Some of the deposits have recurred.
FIG. 4d.—Shows clearing of the deposits after a second course of Provera–100 mg.

1a

1b

2a

2b

2c

3a

3b

Paine, Wright and Ellis.

4a

4b

4c

4d small lung shadows—presumptive metastases—which have not progressed during the year in which she had been on progestogen, nor in the 3 months since the drug has been stopped.

### DISCUSSION

In the male patients with carcinoma of the kidney the main response to progestogen has been in the regression of pulmonary metastases. One patient also had a marked effect on a primary tumour, and in another there was an effect on a malignant pleural effusion. All these responses have occurred within 3 months of starting the drug, and metastases in other tissues—even on the pleural surface—have sometimes progressed while lung deposits were shrinking. In the 6 cases where pulmonary metastases were the primary condition for assessment, 3 responded; where response did occur it was of worthwhile duration—usually over 1 year.

A greater proportion of responses was obtained in the cases of carcinoma of the body of the uterus, and although in 3 of the responding cases it was the pulmonary metastases which demonstrably responded, in one of those with lung deposits pelvic recurrence did not advance, and in Case 7 the peritoneal tumour has perhaps been controlled. In none of these patients has the tumour progressed at one site while regressing at another. Again, response could be shown within 3 months in all cases, and has been of useful duration.

Our limited experience in tumours not of renal or uterine origin does not seem worthy of further comment.

As a result of our observations and of those of others on the use of progestogen in these types of tumour, we feel that the following principles apply. In the patient with asymptomatic metastases, it seems reasonable to allow sufficient time to elapse to obtain an assessment of rate of growth before starting hormone therapy (as by repeat X-rays 1 month after the patient has first been seen). Where symptoms are present from bony metastases, progestogen has not been of use, and we prefer local irradiation. We have used testosterone in 6 renal cases; in 2 instances after the response to progestogen was lost, and in 4 patients who did not respond to it, but no demonstrable response was obtained in any patient. Symptomatic soft tissue metastases, or demonstrably progressing soft tissue metastases likely soon to cause symptoms, are perhaps the indication for progestogen, though our experience suggests that if no response has occurred by the end of 3 months, none is likely to be obtained.

We are indebted to Dr. W. P. Goodyear and Messrs. Upjohn Ltd. for the supply of Provera–100 mg. before this drug became available for general prescription, and to our clinical colleagues for referring the patients for entry into the trial.

### REFERENCES

Bloom, H. J. G.—(1964) 'Hormone treatment of renal tumours: experimental and clinical observations' in 'Neoplastic Disease at Various Sites'; Volume 5: 'Tumours of the Kidney and Ureter' edited by Sir Eric Riches. London (E. & S. Livingstone) p. 311.—(1967) 'Treatment of renal cell carcinoma with steroid hormones: observations with transplanted tumors in the hamster and incurable cancer in man' in 'Renal Neoplasia' edited by J. S. King. Boston (Little, Brown and Co.) pp. 605–633.

Bloom, H. J. G., Dukes, C. E. and Mitchley, B. C. V.—(1964) *Br. J. Cancer*, **17**, 611.

BLOOM, H. J. G. AND WALLACE, D. M.—(1964) *Br. med. J.*, ii, 476.
BRIGGS, M. H., CALDWELL, A. D. S. AND PITCHFORD, A. G.—(1967) *Br. J. Hosp. Med.*, **2**, 63.
HORNING, E. S. AND WHITTICK, J. W.—(1954) *Br. J. Cancer*, **8**, 451.
JENKIN, R. D. T.—(1967) *Br. med. J.*, i, 361.
KIRKHAM, H. AND ROBBINS, M.—(1959) *Natn. Cancer Inst. Monogr.*, **1**, 93.
MATTHEWS, U. S., KIRKHAM, H. AND BACON, R. L.—(1947) *Proc. Soc. exp. Biol. Med.*, **66**, 195.
SAMUELS, M. L., SULLIVAN, P. AND HOWE, C. D.—(1968) *Cancer, N.Y.*, **22**, 525.
SMITH, J. P., RUTLEDGE, F. AND SOFFAR, S. W.—(1966) *Am. J. Obstet. Gynec.*, **94**, 977.